# A survey of current management practices for delayed maculopapular exanthemas during antibiotic treatment among primary care pediatricians

**DOI:** 10.1186/s13052-025-02106-4

**Published:** 2025-08-27

**Authors:** Lucia Liotti, Annamaria Bianchi, Giuseppe Crisafulli, Silvia Caimmi, Paolo Bottau, Fabrizio Franceschini, Rocco Luigi Valluzzi, Sara Riscassi, Francesca Saretta, Michele Miraglia del Giudice, Carlo Caffarelli, Francesca Mori

**Affiliations:** 1 Pediatric Unit, Salesi Hospital, Ancona, Italy; 2 Pediatric Unit, San Camillo Forlanini Hospital, Roma, Italy; 3 Allergology Unit, Pediatric Department, University of Messina, Messina, Italy; 4 Clinica Pediatrica Unit, San Matteo Hospital, University di Pavia, Pavia, Italy; 5 Pediatric and Neonatology Unit, Imola Hospital, Imola, Italy; 6 Private Practice in Allergy, Ancona, Italy; 7 Allergology Unit, Pediatric Department, Bambino Gesu’ Pediatric Hospital, Roma, Italy; 8 Pediatric Department, Bolzano Hospital, Bolzano, Italy; 9 Primary Care Pediatrician, Azienda Sanitaria Universitaria Friuli Centrale, Udine, Italy; 10 Department of Woman, Child and of General and Specialized Surgery, University of Campania ’Luigi Vanvitelli’, Naples, Italy; 11 Department of Medicine and Surgery, Clinica Pediatrica, University of Parma, Parma, Italy; 12 Allergy Unit, Meyer Children’s Hospital IRCCS, Florence, Italy

**Keywords:** Antibiotics allergy, Maculopapular exanthemas, Drug hypersensitivity reaction, Drug allergy, Children

## Abstract

Several guidelines recommended how to manage delayed maculopapular exanthemas during antibiotic treatment. The aim of the present survey was to assess knowledge gaps of primary care pediatricians in managing children with delayed maculopapular exanthemas during a course of antibiotics. Methods: We conducted an online survey among primary care pediatricians in Italy, focusing on the management of children with maculopapular exanthemas occurring during antibiotic administration. Results: We found that 41% of pediatricians continued with the same antibiotic after the onset of mild to moderate maculopapular exanthemas. Additionally, only 25% took pictures of the skin manifestations during the acute phase, and 66% recorded the latency of the reaction. Conclusions: Primary care management of children with suspected antibiotic induced maculopapular exanthemas is heterogeneous. Primary care physicians and allergists need to share common decisions and protocols to avoid mislabelling children as allergic to antibiotics.

## Background

Maculopapular exanthema (MPE) is the most frequently reported adverse drug reaction (ADR) in children [1]. MPE is commonly classified as a delayed reaction when it occurs after 6 h from the last drug intake. The mechanisms underlying MPE are not fully elucidated even in the recent classification by Jutel M et al. [2] T lymphocytes are the effector cells activated in the immune response and the reaction was categorized as a Type IVb hypersensitivity reaction according to Gell and Coombs’s classification [3]. MPE can occur through both hapten- [often with beta-lactams (BL)]or p-i mechanisms [4]. The latter can explain a stimulation of T cells, without the involvement of innate immunity as in Drug Reaction with Eosinophilia and Systemic Symptoms (DRESS). MPE are characterized by itchy erythematous macules and infiltrating papules predominantly affecting the trunk and proximal parts of upper and lower arms, in a symmetrical distribution. Mucosal involvement is often absent. The interval before the onset varies from 4 to 14 days from the beginning of treatment [5]. BLs are the most frequently involved drugs in hypersensitivity reactions in children [1]. It has been shown that 5–10% of children treated with amoxicillin develop MPE during the course therapy [6]. MPEs are classified based on their clinical presentation as mild, moderate or severe [7]. Most MPEs are mild to moderate in severity. Differentiating between a drug hypersensitivity reaction and a viral exanthema is often challenging and may even be impossible if only skin lesions are considered. Moreover, most of MPEs are not confirmed by a drug allergy workup. Mild MPE occurring during an antibiotic treatment, is the result of an immune-mediated drug hypersensitivity reaction in only 5–10% of cases [6]. Physicians must be aware that most MPEs occurring during antibiotic treatments are not due to drug hypersensitivity but are due to the concurrent viral illness [8]. Consequently, many children are incorrectly labeled as allergic to drugs, mainly BLs or non-steroidal anti-inflammatory drugs. On the other hand, MPE may be part of more severe reactions such as severe cutaneous adverse reactions (SCARs). It is of note that MPE may represent the first sign of DRESS [9, 10] and physicians should promptly recognize such a severe evolution. Physicians should know the danger signs (i.e., fever > 38.5 °C, mucosal or organ involvement, bullous eruptions, eosinophilia, leucopenia, hypocomplementemia, etc.) to remove the trigger as soon as possible [5]. Moreover, it is reported that the two conditions (drug hypersensitivity and viral infection) may coexist, and the viral infection may facilitate the occurrence of a persistent state of drug hypersensitivity. This condition is most frequently described in case of moderate to severe MPE during Epstein Barr Virus Infection and concomitant amoxicillin use [11, 12]. In general, on one side the labeling should be accurately removed and, in most instances, pediatrician could directly reuse the suspected BL, particularly in mild MPE cases, where the clinical manifestations are probably not caused by drug hypersensitivity and the chance of future reaction is very low. Conversely, suspected drug hypersensitivity should be thoroughly investigated to prevent potential future reactions. The diagnostic and treatment approaches for delayed MPE vary among physicians [7]. We undertook a survey to explore practice patterns of primary care pediatricians in managing children with MPE during antibiotic treatment.

## Methods

The Drug Allergy Committee of the Italian Pediatric Society of Allergy and Immunology (SIAIP) developed a 12 items survey (Table 1). The survey was circulated to primary care pediatricians across Italy. The survey was disseminated through regional leaders of the Italian Federation of Pediatricians (FIMP) and regional coordinators of SIAIP via local channels and it potentially reached approximately 4000 pediatricians. The on-line survey was filled out, from January to April 2024. It included data of children aged 0–14 years. The criteria provided to pediatricians to identify the severity of MPE to antibiotics were those of Romano et al. [7]. A mild MPE is characterized by skin eruption involving less than 50% of body surface area, lasting less than one week, with no systemic involvement. A moderate MPE involves a skin eruption affecting > 50% of body surface area, lasting more than one week, with no systemic involvement. A severe MPE is defined by a skin eruption involving > 50% of body surface area, with infiltration, confluent and erythrodermic areas, lasting more than one week, and accompanied by systemic involvement such as fever, eosinophilia, lymphadenopathy, or organ damage. Vesicles or pustules are less frequent manifestations of severe MPEs. The survey consisted of 12 questions. Four questions were focused on characterizing primary care pediatricians, inquiring their region of practice, years of experience, and the number of patients with suspected delayed MPEs to antibiotics they have evaluated in the last years. The remaining 8 questions were focused on the clinical, practical and therapeutic management of children with suspected delayed MPEs to antibiotics. Each question had 4 answer options, with only one choice permitted (Table 1).

**Table 1:** Questionnaire for primary care pediatricians

	QUESTIONS	ANSWERS
**1**	**How many patients with suspected mild-moderate delayed skin reaction to antibiotics have you evaluated in the last years?**	• 0 • 5–10 • 11–20 • > 20
2	**How many patients with suspected mild-moderate delayed skin reaction to antibiotics have you evaluated in the last 5 years?**	• 0 • 5–10 • 11–20 • > 20
3	**If one of your patients has a suspected mild-moderate delayed skin reaction over the course of antibiotic treatment**,** do you usually…. (several answers are possible)**	• I take photos of the lesions • I assess and note the extent of the lesions and the presence of itching • I note the timing of the reaction in relation to the taken drug • I note the recommended therapy, the potential discontinuation of the drug and its replacement, the patient’s tolerance to it • I re-evaluate the patient after 24 h
4	**Scenario: a patient shows a suspected mild-moderate delayed skin reaction to antibiotics during a febrile illness. Which one of these answers corresponds mostly to your behavior?**	• I immediately stop the suspected drug • I immediately stop the suspected drug and recommend antihistamines • I immediately stop the suspected drug and recommend antihistamines and/or corticosteroids •I do not interrupt the suspected drug, and I re-evaluate the patient shortly •I do not interrupt the suspected drug, and I recommend antihistamines and/or corticosteroids and re-evaluate the patient shortly
**5**	**If you stop the suspected drug but you need to continue an antibiotic therapy…**	• I choose a different class of antibiotics (e.g., switch from amoxicillin to macrolide) • I choose a drug belonging to the same class, but of a different category (e.g., switch from amoxicillin to cephalosporin) • I choose a different formulation maintaining the same active ingredient (stop suspension and choose tablets or granules) • I do not prescribe any antibiotics and send the patient to the Emergency Room
**6**	**If a patient with a suspected mild-moderate delayed skin reaction to antibiotics needs the same antibiotic in the future**	• I try to administer the suspected drug again at home • I try to administer the first dose of the suspected drug again in my clinic • I try to administer the suspected drug again and prescribe antihistamines at the same time • I choose a different class of antibiotic (e.g., macrolide instead of amoxicillin) • I choose a drug belonging to the same class, but to a different category (e.g., cephalosporin instead of amoxicillin)
**7**	**If you have chosen the first or second answer to question 6 (I try to administer the suspected drug again)**,** in your experience**	• The patient has always tolerated the suspected drug • The patient has often tolerated the suspected drug • Approximately 50% of patients tolerated the suspected drug • The patient has rarely tolerated the suspected drug
**8**	**If one of your patients has a suspected mild to moderate delayed skin reaction to antibiotics**,** do you refer him/her to the allergists?**	• I always require an allergy assessment • I never require an allergy assessment • I require an allergy evaluation if I had discontinued the antibiotic and replaced it with another molecule • I require an allergy evaluation if the patient has had the same skin reaction on several occasions or with multiple drugs • I require an allergy evaluation if the patient has a family history of drug allergy
**9**	**If you prescribe an allergy assessment**,** how long should it take to carry it out after the suspected antibiotic reaction?**	• Within 1 month • Within 3–6 months • Within 1 year • It depends on the age of the child
**10**	**One of your patients has a suspected mild to moderate delayed skin reaction to antibiotics and undergoes a complete allergy evaluation with a negative drug provocation test. What is your behavior?**	• I will prescribe the same drug again, if needed in the future •I prefer to administer other antibiotics anyway •I leave the choice to the family
**11**	**How long have you been practicing as a pediatrician?**	• *≤*5 years • 6–20 years • 21–30 years • >30 years
**12**	**In which region do you perform your profession?**	

## Results

The questionnaire was filled out by 553 pediatricians from 17 out of 20 Italian Regions: 35,3% from North Italy, 38.9% from Central Italy and 25,8% from South Italy and Islands.

72% of pediatricians had more than 20 years of practice as primary care pediatricians and only 5.4% of participants had less than 5 years of work experience. 53% of participants reported to see about 5–10 children per year with delayed mild-moderate MPE over the course of antibiotic (mainly BL) treatment. Moreover, in the last 5 years, 21% of pediatricians managed about 11–20 children with such a type of reaction and 14.8% of participants evaluated more than 20 patients. In case of suspected MPEs occurring during antibiotic treatment, pediatricians followed different approaches to manage patients: only 25% of general practitioners were used to take a picture of the skin lesions; 47% of them used to assess the extension and the presence of itching; 66% used to ask about the time latency and 48.5% used to follow up patients with acute MPE by performing an “in person” visit after 24 h. In case of MPEs occurrence during fever and concomitant antibiotic treatment: 58,2% of primary care pediatricians interrupted the antibiotic treatment and 41.8% continued to treat the patient with the same antibiotic, shortly re-evaluating the patient. Among those who interrupted the drug: 52% prescribed antihistamines and 32.8% prescribed both antihistamines and corticosteroids. Of those who continued the antibiotic treatment, 61.7% added antihistamines.

**Fig. 1:**
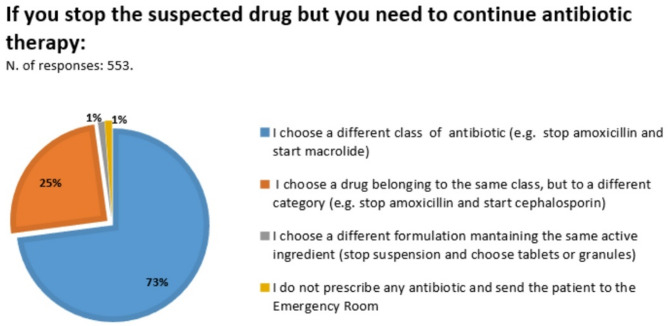
Answers to question 4

When the antibiotic was stopped and it was necessary to treat the disease with an alternative drug, 72.9% of participants switched to a different antibiotic class (i.e., macrolides); 24.9% of participants chose an antibiotic belonging to the same class (i.e. a third-generation cephalosporin was most commonly prescribed when a MPE occurred during amoxicillin/clavulanic acid treatment and vice versa) (Fig. 1).

In case of future necessity of the same BL antibiotic that provoked a mild-moderate MPE: 50.4% of participants prescribed a different class of antibiotic (i.e. macrolides); 28% choose a different antibiotic of the same class (for example a cephalosporin instead of amoxicillin); 21.2% of pediatricians prescribe again the same molecule (11.2% at home, 4.9% by administering the first dose in an out-patient setting, 5.1% combining the antibiotic with anti-histamine).

The 131 primary care pediatricians who have prescribed the same molecule, for example a BL that provoked a mild MPE, stated that 26,7% of patients always tolerate the same antibiotic, 67,9% of patients often tolerate the same antibiotic and 5.3% of patients rarely tolerate the same antibiotic.

**Fig. 2:**
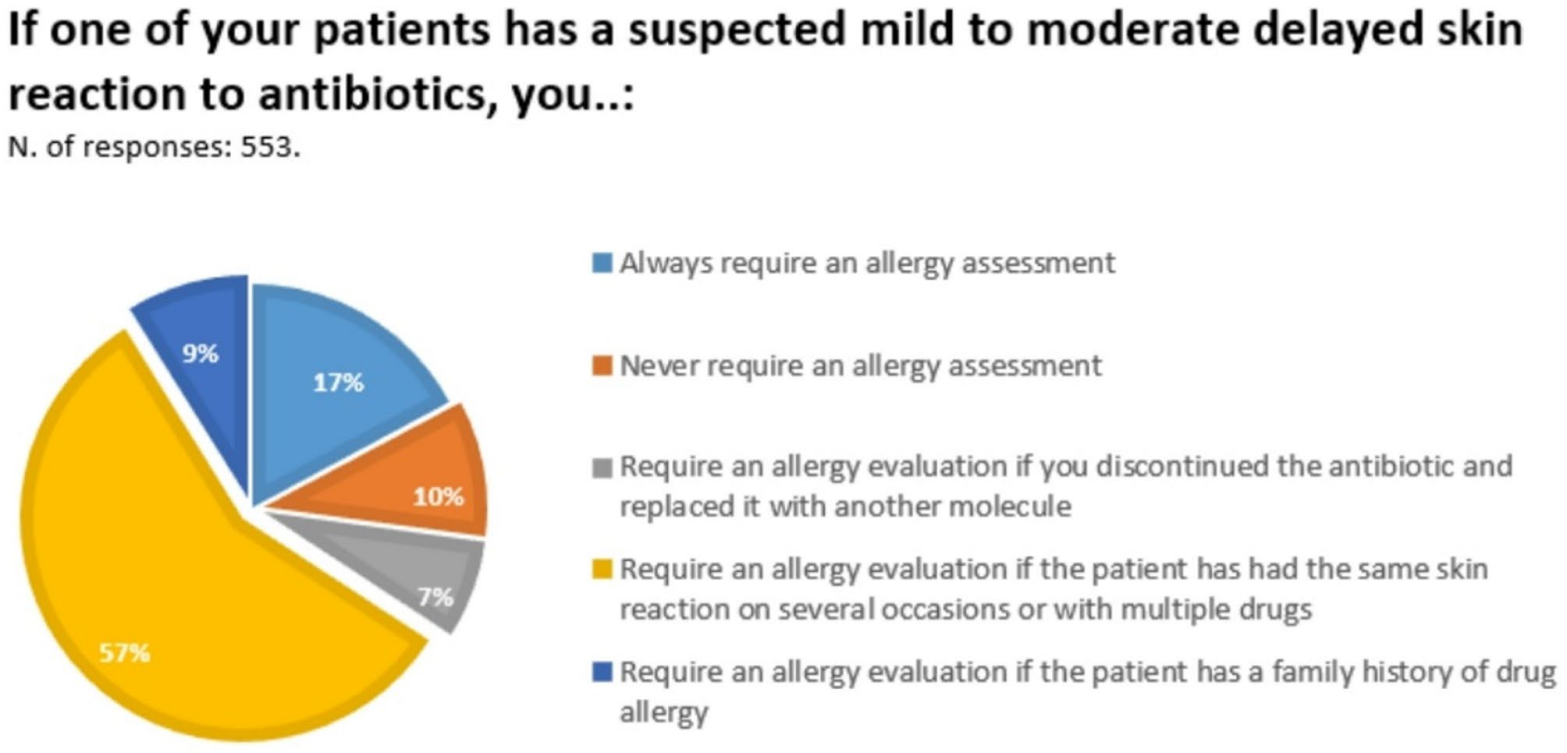
Answers to question 8

In case of delayed mild-moderate MPE during BL treatment, more than half of participants (57%) referred patients to the allergist only in case of recurrent reactions with the same molecule or with a different one. Patients were always referred to the allergist by 17.2% of participants, by 8.9% only in case of family history of drug allergy, by 7.1% only in case of switch to a different molecule. It is noteworthy that 9.9% of participants never referred those patients to the allergist (Fig. 2).

Regarding the timing for referral, 42.8% of participants think that the allergy work-up should be performed within 3–6 months from the reaction; 28% within a month, 10.3% within 12 months and 19% think that the timing for allergy evaluation should be adapted to the age of patient.

When a complete allergy work-up including a negative drug provocation test showed that the suspected drug was tolerated, 81% of participants feel confident in prescribing again the same antibiotic while 16.6% of participants would not prescribe again the drug in question and 1.3% of participants leave the decision to the family.

## Discussion

In the present study it was found that, in children with mild to moderate MPEs during antibiotic treatment, only a few pediatricians continue with the same antibiotic, while in most cases the medication is switched to a different one. This management is in agreement with the common practice adopted by European physicians. In the United States, the approach consists of continuing with the administration of the same drug in case of mild reactions [13].

Two key issues resulted from the survey. In the digital selfie era, only a small percentage (25%) take a picture of the skin manifestations in the acute phase and not all pediatricians (66%) record the latency of the reaction. It is important for pediatricians to modify these behaviors as both informations are extremely useful to assess the level of risk for each patient and to choose the appropriate diagnostic pathway. The recent findings promote a personalized drug allergy work up to facilitate the diagnosis in terms of tolerance and timing and to reduce the risk for the patient ^**11**^. Both the pediatric task force and the adult guidelines suggest you directly provoke children with a history of delayed mild MPE [6, 14]. In most cases, those children are at low risk of developing again a MPE during antibiotic treatment. Therefore, recent studies suggest re-administering the same antibiotic in an outpatient setting [15]. It could be considered useful to modify the formulation of the medication to reduce the risk of reactions to the additives [16]. However, this survey shows that only a small proportion of Italian pediatricians (21.2%) prescribe the same molecule again. This rate is in agreement with other studies including only children and both children and adults. Prematta T et al. [17] found that a small number of providers were comfortable in prescribing penicillin (9.7%) or amoxicillin (15%) to a patient with a history of MPE to these drugs. This percentage increased to 25% in case of penicillin and to 35.7% in case of amoxicillin if prescriptions came from allergists [16]. Aligned with the present study were also the results of a Thai survey conducted by Suetrong N et al. [18]. This study compared the management of patients with a history of penicillin allergy between allergists and non-allergists showing that the majority of both allergists (65.5%) and non-allergists (63.6%) would have avoided only penicillin in patients with a history of penicillin-induced MPE [18]. In the era of antibiotic resistance, it is important to treat infectious diseases with the most proper antibiotics to avoid complications, excessive costs, and prolonged hospitalization. Consequently, it is of paramount importance to educate primary care pediatricians in referring patients to the allergists when they are not confident in re-prescribing the antibiotics suspected to be involved in delayed mild MPE.

Another important point is the reliability of the results obtained from the allergy work-up [19]. Our survey shows that allergy investigations increased the percentage of reuse of the suspected drug from 22.2 to 81%. So, it is surprising that there are pediatricians who are still not confident in prescribing the antibiotic again even if it was tolerated as ascertained by a negative drug provocation test outcome. Accurate information must be provided to pediatricians to reduce the number of those who remain unconvinced. The authors of the present study suggest to weight out risks-benefits for each patient and to threating trough those children who develop a mild MPE over a course therapy. On the other hand, for those who require an antibiotic switch, if the pediatrician is not confident in re-prescribing the same drug in the future a complete drug allergy work up should be guaranteed within a 6 months a year in order to remove the label of drug allergy.

## Conclusions

Primary care pediatricians play a key role in the management of children with delayed MPE during antibiotic treatment and concomitant infectious disease. Our survey shows that the primary care management of children with suspected antibiotic induced mild MPE is heterogeneous in Italy. It is important to enhance pediatricians’ understanding of the true risks associated with drug hypersensitivity, in order to prevent unnecessary withdrawal of the suspected medication and to avoid mislabelling children as allergic to antibiotics. Due to the risks associated with antibiotic resistance, prevention of labeling should be a shared goal. In this regard, primary care physicians and allergists need to share common decisions and protocols. It is important that families are informed about the final decision, considering the risks and benefits for the patient. However, the decision must not be made by the families to ensure that choices are based on the best interest of the patient.

## Data Availability

Data sets generated during the current study are available from Lucia Liotti on reasonable request.

## Abbreviations

MPE: Maculopapular exanthemas;
ADR: Adverse drug reaction;
BL: Beta-lactams;
DRESS: Drug Reaction with Eosinophilia and Systemic Symptoms;
SCARs: Severe cutaneous adverse reactions;
